# Comparative morphology of the cruciate ligaments: A radiological study

**DOI:** 10.1515/med-2024-1005

**Published:** 2024-07-29

**Authors:** Xin Gan, Xin Chen, Yunqian Zeng, Mengwei Li, Mingbo Nie, Hao Kang

**Affiliations:** Department of Orthopedic, Tongji Hospital, Tongji Medical College, Huazhong University of Science and Technology, Wuhan, Hubei, P. R. China; National Medical Center for Major Public Health Events, Wuhan, P. R. China

**Keywords:** cruciate ligament, ACL, PCL, knee joint, MRI, correlation

## Abstract

**Background:**

The anterior cruciate ligament (ACL) and posterior cruciate ligament (PCL) are important structures to maintain knee stability. The present study aimed to further enrich understandings of the morphology of the cruciate ligaments and explore the relationship between the diameter of ACL and PCL.

**Method:**

This study collected valid MRI samples of 50 male and 50 female normal right knee joints and measured the diameter of each point of the ACL and PCL through the 3D Slicer.

**Results:**

The diameter of the ACL in the sagittal MRI of the normal right knee joint was significantly different from the diameter of each point of the PCL. The average diameter of each point of the ACL was larger than the diameter of the corresponding point of the PCL. Males and females had statistical differences in their PCL origin point, PCL midpoint, ACL origin point, ACL midpoint, and ACL insertion point diameters under sagittal MRI examination. The average diameter of males was greater than the average diameter of females at the above corresponding sites. In sagittal MRI scans of the normal right knee joint, we observed that only the origin point of the PCL exhibited a moderate correlation with the midpoint and insertion point of the ACL in terms of their respective diameters.

**Conclusion:**

The correlation between diameters of normal ACL and PCL in knee joint MRI was moderate and may help clinicians determine appropriate graft for cruciate ligament reconstruction surgery quickly for severe cruciate ligament injuries.

## 1 Introduction

The knee joint, pivotal for weight-bearing and mobility, is central to everyday activities such as walking and stair climbing. Cruciate ligaments within the knee are instrumental in maintaining its stability [1]. A growing body of medical research underscores the significance of graft cross-sectional area selection in knee cruciate ligament reconstruction surgery. Such a choice can profoundly influence postoperative knee function recovery and graft longevity [2–5]. A multitude of studies delve into the cross-sectional area and anchoring positions of the anterior cruciate ligament (ACL) and posterior cruciate ligament (PCL) on the femur and tibia [6,7]. Given that contemporary cruciate ligament reconstruction predominantly employs the patient’s autologous ligament as a graft [8], the patellar and hamstring tendons emerge as the preferred graft choices [8,9]. Against this backdrop, researchers are intensifying efforts to accurately measure both the cross-sectional area of the ligament set for reconstruction and the suitable autologous ligament [2–5].

The employment of computerized three-dimensional software offers a nuanced lens for evaluating cruciate ligament morphology, aiding clinicians in their preoperative graft selection decisions [10]. MRI, an emergent radiological technique, has garnered widespread acceptance, particularly in developed regions, for knee joint examinations. This modality has enabled researchers to assess the cross-sectional area of various knee structures [11]. Several studies have leveraged MRI to gauge the cross-sectional areas of the hamstring or semitendinosus tendons, facilitating more precise autologous tendon graft choices for ACL reconstruction surgeries [2,11]. While MRI-based anatomical studies of the knee’s ACL and PCL are not rare, comparative investigations focusing solely on the diameters of the ACL and PCL using MRI remain strikingly scant.

MRI is an indispensable tool in the clinical evaluation of the knee joint. By assessing the diameter of the ACL and PCL using MRI, clinicians can gain a more profound insight into the imaging characteristics of these ligaments in their normal state. This knowledge is particularly salient when dealing with significant injuries or intense physical activities that may compromise either the ACL, the PCL, or both. Such severe injuries can lead to a segmental loss of these ligaments, rendering cross-sectional measurements unfeasible. Therefore, we speculate whether the diameter of the residual injured cruciate ligament, or the diameter of the intact ACL or PCL, can be utilized as indicators for inferring and assessing the diameter of another severely damaged cruciate ligament.

This study aims to elucidate the relationship between ACL and PCL diameters in knee MRI scans to enhance clinical understanding and inform cruciate ligament reconstruction surgery decisions. By examining these morphological ties, clinicians can optimize graft selection in cruciate ligament reconstruction surgeries, with the hope of improving patient outcomes. In this investigation, we utilized state-of-the-art knee MRI images sourced from the NYU School of Medicine Langone Health fastMRI database [12] to delineate the interrelationship between the ACL and PCL in MRI diagnostics.

## 2 Materials and methods

### 2.1 Data source and study population

This study entailed a retrospective analysis of anonymized radiographic data sourced from the public domain. The imaging corpus for this endeavor was derived from the NYU Langone Health fastMRI [12], a repository maintained by the Center for Advanced Imaging Innovation and Research within the Department of Radiology at NYU School of Medicine and NYU Langone Health. This repository encompasses a spectrum of sub-datasets, including raw k-space data. We uniformly selected MRI images of the right knee joint for analysis.

For this investigation, Digital Imaging and Communications in Medicine (DICOM) metadata from subjects within the NYU Langone Health fastMRI database [12], supplied by NYU Langone, were meticulously selected based on specific inclusion criteria. We incorporated individuals aged between 20 and 60 who exhibited normal right knee joint function, presented no conspicuous history of ACL and PCL injuries, and had not undergone knee surgeries. Exclusion criteria encompassed subjects: (1) exhibiting congenital abnormalities or absent/partially missing ACL and/or PCL; (2) where the origin, midpoint, and insertion points of the ACL and PCL were indiscernible; and (3) presenting torn or inflamed ACL and PCL.

The dataset under investigation was parsed to distinctly classify subjects based on gender into male and female cohorts. Subsequent to this demarcation, 88 DICOM metadata entries were indiscriminately chosen from each cluster, aggregating to 176 selected entries for this research. These entries, when processed via 3D Slicer, were meticulously assessed with a spotlight on Sagittal Proton Density with Fat Suppression (SAG PD FS) images, integral for comprehensive MRI analysis. Of the original 176 DICOM metadata entries, 175 passed the inclusion protocol. However, upon application of the exclusion criteria, 71 of these were deemed unsuitable and were subsequently removed. Delving into specifics, 48 were excluded due to discernible injuries to the ACL and/or PCL, evidenced by inflammation, mutation, or outright tears. Meanwhile, the origin, midpoint, and insertion of the ACL and PCL were ambiguous in 23 cases, leading to their exclusion. The refined dataset comprised 50 male and 54 female entries. However, to institute a balanced comparative analysis between male and female ACL and PCL relationships, the clearest 50 entries from each gender were earmarked for precise measurement. It is pivotal to note that the MRI imagery for this study was sourced from unidentified, fully sampled knee MRI datasets courtesy of NYU Langone. Further technical specifics reveal that the deployed 3.0 Tesla MRI scans were characterized by sagittal proton density with an SAG PD FS, a parameter tailored for imaging normally functioning knee joints.

### 2.2 Measurement points of the cruciate ligaments and their appearance under MRI images

Image processing, analysis, measurement, and collection were performed using 3D Slicer version. The ACL and PCL of the right knee joint were found through the SAG PD FS images of the subjects using the 3D Slicer version. For this project, to study the relationship between the diameter of the ACL and the PCL of the right knee joint, three representative positions of the ACL and PCL (origin point, midpoint, and insertion point of the cruciate ligament) were selected for measurement.

**Figure 1:**
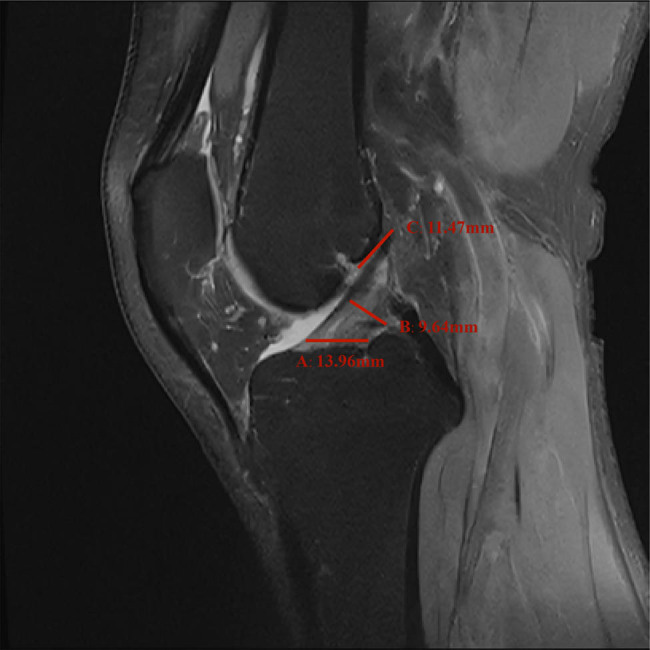
Schematic picture of the diameter measurement of each point of the ACL: A represents insertion point, B represents midpoint, and C represents origin point of the ACL.

In the sagittal MRI, the ACL originates on the medial surface of the lateral femoral condyle, passes antero-inferiorly, and terminates at the bone surface in front of the tibial intercondylar bulge. The normal ACL in the MRI of the knee joint appears as a relatively loose mid-to-low signal shadow. There is a line-like, stripe-like medium or high shadow separation at its attachment. The origin point of the ACL was selected to be measured obliquely below the posterior part of the lateral femoral condyle of the ligament. The midpoint diameter of the ACL was measured at the midpoint of the ACL perpendicular to the cruciate ligament. The insertion point of the ACL measured was selected to be 2 mm above the bone surface in front of the tibial intercondylar bulge. Figure 1 shows a schematic diagram of the measurement of the diameter of the ACL.

**Figure 2:**
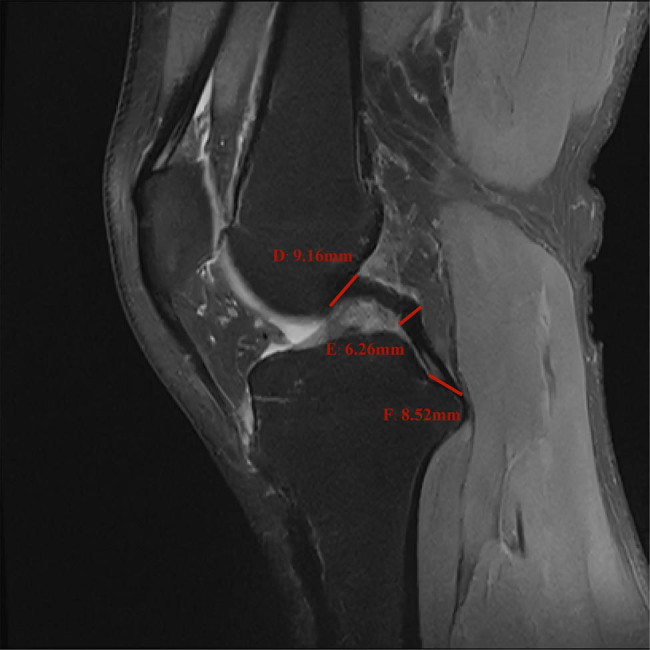
Schematic picture of the diameter measurement of each point of the PCL: D represents origin point, E represents midpoint, and F represents insertion point of the PCL.

In the sagittal MRI, the PCL originates from the lateral surface of the medial femoral condyle, passes posteriorly, and terminates at the articular surface of the posterior tibia. The normal PCL in the knee joint MRI image shows low signal intensity. The sagittal PCL was arched convexly backward, with smooth edges, and at an angle of approximately 40–50° with the tibial plateau [13–15]. The origin measurement point of the PCL was chosen to be 2 mm below the lateral surface of the medial femoral condyle. The midpoint measurement of the PCL was selected to be the convex point of the PCL in the sagittal MRI of the kyphotic arch, and the insertion point of the PCL was selected as the point where the ligament inserts 2 mm above the articular surface of the posterior tibia. Figure 2 shows a schematic diagram of the measurement of the diameter of the PCL.

The DICOM metadata package MRI provided in the NYU Langone Health fastMRI database mainly includes three parts: T1 weighted, T2 weighted, and proton density weighted with fat suppression. The T1 weighted image can be regarded as the mapping expression of the proton energy in human adipose tissue, and it is an image that highlights human adipose tissue [12]. The T2 weighted image is a representation of the proton energy map in the fat- and water-based tissues of the human body. By comparing with the T1 weighted image, the adipose tissue can be distinguished from the water-based tissue. Therefore, what is bright on the T2 image but dark on the T1 image is the liquid tissue in the human body. However, for images that are proton density weighted with fat suppression, when imaging human tissues, long repetition time [1] (2,000 ms) and short echo time [8] (30–40 ms) sequences are often selected for proton-density-weighted imaging. Because the long TR and short TE can reduce the influence of T1 and T2 on the imaging signal, this can highlight the proton density-related signals on the image. At the same time, fat suppression can better observe the ACL and PCL. Moreover, data analysis under the same series of MRIs can minimize the measurement error. Therefore, the measurement points that were selected (i.e., origin point, midpoint, and insertion point) of the knee joint ACLs and PCLs were all analyzed and measured under the subject’s SAG PD FS images.

### 2.3 Reliability and repeatability of the project

Researchers were trained in standardized cadaver dissection and imaging interpretation training. Proficient in using 3D Slicer software to segment and measure MRI parts accurately. Multiple repetitive tests were performed for each measurement, taking an average of an hour per set of data. All measurements in this study were independently conducted by two experienced orthopedic doctors, both adhering to the same standardized procedures. Subsequently, the average value derived from their independent assessments was utilized for analysis. The measured data underwent statistical analysis conducted by a third researcher.

### 2.4 Dataset and statistical analysis

Utilizing IBM SPSS (Version 22, Armonk, NY, USA), we administered both the Kolmogorov–Smirnov (K–S) and Shapiro–Wilk (S–W) tests to ascertain the normality of our dataset. Data adhering to a normal distribution were characterized by the mean ± standard deviation (SD). To discern differences across datasets, we employed the independent sample *t*-test, whereas the paired *t*-test was reserved for analogous measurement points. For data abiding by a normal distribution, the Pearson correlation analysis was invoked. Correlation strengths were delineated as follows: a coefficient ≥0.8 indicated a strong correlation, a coefficient between 0.5 and 0.8 signified a moderate correlation, a coefficient from 0.3 to 0.5 represented a low correlation, and a coefficient below 0.3 suggested an exceedingly weak correlation, verging on irrelevance. A *p*-value threshold of <0.05 was set as the marker for statistical significance.


**Ethical approval:** The study was conducted according to the principles of the Helsinki Declaration. The approval of the study from the ethics review board was waived because the research relied upon publicly used, de-identified secondary data.

## 3 Result

The measurement values of the diameter of the ACL and PCL of the right knee joints of a group of 100 valid subjects (including 50 men and 50 women) were obtained. There were six groups in total of males and females: the diameters of the origin point, midpoint, and insertion point of the ACLs, and the diameters of the origin point, midpoint, and insertion point of the PCLs (Table 1). The units of measurement data are in millimeter [16]. Comparison was carried out as to whether the ACLs and PCLs are related in terms of diameter, comparing the size of the ACLs and PCLs at each point, and whether the male and female results are related in terms of the ACL and PCL measurements.

**Table 1:** Diameter of the ACL and PCL^a^

	Diameter (male, *N* = 50)	Diameter (female, *N* = 50)
PCL origin (mm)	9.83 ± 0.88	8.58 ± 0.88
PCL middle (mm)	6.54 ± 0.57	6.27 ± 0.52
PCL insertion (mm)	9.16 ± 0.68	8.99 ± 0.56
ACL origin (mm)	11.74 ± 0.82	11.31 ± 0.78
ACL middle (mm)	9.59 ± 0.61	8.08 ± 0.47
ACL insertion (mm)	12.68 ± 0.89	10.69 ± 0.71

^a^The values are shown as the mean ± SD. The units of measurement data are mm.

### 3.1 Analysis of the difference of the starting point, midpoint, and end point diameter of the ACLs and PCLs

The K–S test and S–W test were performed on the normal distributions of these six sets of data (starting point diameter, midpoint diameter, and endpoint diameter of the ACL and PCL), they all conformed to a normal distribution. According to the statistics and analysis results obtained in Table 2, by conducting *t* tests on the origin point diameter, midpoint diameter, and insertion point diameter of the ACLs and PCLs, the *p*-values of the three groups of differences were all less than 0.05. Therefore, there were significant statistical differences in the *t* tests for the diameters of these points of the PCL and ACL. From the mean and range values of each site, it can be concluded that the diameter of the ACL was greater than the diameter of the PCL at each of the given points.

**Table 2:** Simple Student’s test combining male and female data^a^

Segment	PCL (*N* = 100)	ACL (*N* = 100)	*t*	*p*
Origin	9.21 ± 1.08	11.53 ± 0.83	−19.067	<0.001
Middle	6.40 ± 0.56	8.84 ± 0.93	−25.450	<0.001
Insertion	9.07 ± 0.63	11.68 ± 1.28	−20.793	<0.001

^a^The values are shown as the mean ± SD. *t* means simple Student’s test, *p* means *p*-value. The units of measurement data are mm.

### 3.2 Comparison of the difference of the diameter of each point of the ACL and PCL between males and females

Using SPSS, the 12 sets of data that had passed the K–S and S–W normal distribution tests were differentiated using sexual testing. The difference between male data and the female data at the same site was detected using *t* tests, and the mean and SD of each group of data were counted and analyzed. As shown in Table 3, the *p*-values from the results of the *t* tests on the same site of the five sets of data for males and females, i.e., PCL origin point, PCL midpoint, ACL origin point, ACL midpoint, and ACL insertion point were all less than 0.05. Therefore, the diameters of the PCL’s origin point and midpoint, and the ACL’s origin point, midpoint, and insertion point were all statistically different between males and females. From a numerical point of view, the diameters of the male PCL origin point, the PCL midpoint, the ACL origin point, the ACL midpoint, and the ACL insertion point were all higher than those of females at the same site. The *p*-value was found to be 0.187 when the *t* tests were performed on the male and female groups of the PCL insertion point diameter. As the *p*-value of this group was greater than 0.05, the difference between males and females at the point of the PCL’s insertion point diameter was not statistically significant.

**Table 3:** *t*-test for each group of males and females^a^

	Male (*N* = 50)	Female (*N* = 50)	*t*	*p*
PCL origin	9.83 ± 0.88	8.58 ± 0.88	7.107	<0.001
PCL middle	6.54 ± 0.57	6.27 ± 0.52	2.456	0.016
PCL insertion	9.16 ± 0.68	8.99 ± 0.56	1.330	0.187
ACL origin	11.74 ± 0.82	11.31 ± 0.78	2.656	0.009
ACL middle	9.59 ± 0.61	8.08 ± 0.47	13.792	<0.001
ACL insertion	12.68 ± 0.89	10.69 ± 0.71	12.382	<0.001

^a^The values are shown as the mean ± SD. *t* means *t*-test, *p* means *p*-value. The units of measurement data are mm.

### 3.3 Correlation between the ACL and PCL

As the datasets of each point in the male and female groups had passed the K–S and S–W normal distribution tests, and the six sets of data meet the normal distribution conditions, the correlation of each point diameter index was adopted for Pearson correlation analysis. *p* < 0.05 and *p* < 0.01 were identified as statistically significant. As shown in the results in Table 4, the correlation coefficient between the PCL origin point and ACL midpoint was *r* = 0.517, *p* < 0.05; and the correlation coefficient of the PCL origin point and the ACL insertion point was *r* = 0.615, *p* < 0.05. For the others, the correlation coefficient of each point of the PCL and the ACL was less than 0.3, and *p* < 0.05. In sagittal MRI scans of the normal knee joint, we observed that only the origin point of the PCL exhibited a moderate correlation with the midpoint and insertion point of the ACL in terms of their respective diameters, and those of the diameters of the other points were extremely weak and can be regarded as irrelevant. The correlation coefficient between the PCL origin point and PCL midpoint was 0.505, and *p* < 0.05, there was a positive but weak correlation between them.

**Table 4:** Correlation test of each point diameter of the PCLs and ACLs

	PCL origin	PCL middle	PCL insert	ACL origin	ACL middle	ACL insert
PCL origin	1					
PCL middle	0.505^**^	1				
PCL insert	0.399^**^	0.377^**^	1			
ACL origin	0.202^*^	0.205^*^	0.174	1		
ACL middle	0.517^**^	0.254^*^	0.246^*^	0.320^**^	1	
ACL insert	0.615^**^	0.260^**^	0.287^**^	0.322^**^	0.805^**^	1

*p < 0.05; **p < 0.01.

## 4 Discussion

Our study identified a nuanced relationship between the diameters of the normal ACL and PCL in sagittal MRI examinations of the right knee. Notably, a distinct correlation emerged solely at the origin point diameter of the PCL, exhibiting a moderate relationship with the midpoint and insertion point diameters of the ACL. Remarkably, correlations with other points waned to a negligible degree. Diametric comparisons between ACL and PCL in MRI scans revealed significant disparities. On average, every ACL point diameter surpassed its corresponding PCL counterpart. Gender differences presented themselves with statistical significance across various ligament points in sagittal MRI examinations of the right knee joint: the average diameter for males consistently exceeded that for females at analogous ligament locales, consistent with previous research [17]. Yet, this gender distinction proved inconsequential in the PCL insertion point diameter dataset. These findings resonate, albeit with variations, with preceding research. A cadaveric analysis posited that the ACL and PCL widths in females are comparatively slender than in males [18]. Further, this anatomical inquiry suggested an average ACL width of 6.4 ± 1.4 mm juxtaposed against an average PCL width of 10.2 ± 2.0 mm [18], a conclusion seemingly at odds with our study which determined a more prominent average diameter across ACL points than the PCL.

These discordances can be attributed primarily to two factors. First, the aforementioned cadaveric study [18] gauged the ACL and PCL widths in the coronal plane, whereas our research employed the sagittal plane. Second, the irregularly shaped cross-sections of the ACL and PCL [19] exerted influence on the findings. Typically, the PCL showcases its maximal width in the coronal plane, while the ACL tends to be broader in the sagittal plane [19]. Acknowledging these measurement orientations, coupled with the inherent morphology of the cruciate ligaments, our study’s outcomes align closely with, and are robustly corroborated by, the referenced cadaveric anatomy investigation.

In cruciate ligament reconstruction, the cross-sectional area of each ligament segment stands as a pivotal metric for graft selection [16,20,21]. Integrating our project’s data with traditional anatomical insights on the cross-sectional dimensions and morphology of the ACL and PCL could revolutionize the utilization of MRI scans, enabling accurate determinations of these ligaments’ cross-sectional areas within the patient’s knee. Such synergy promises clinicians enhanced precision in preoperative evaluations of the cruciate ligament’s cross-sectional area, facilitating optimal graft selection. Furthermore, this amalgamated understanding deepens our insights into the radiographic depictions of ACL and PCL diameters, equipping clinicians to adeptly discern these ligaments’ diameters even in pathological joints and pinpoint elusive lesions. This proficiency becomes especially pivotal when confronted with cases of acute cruciate ligament trauma, where direct measurements of the injured ligament’s cross-sectional area may be untenable. Under such scenarios, the remnant diameter of the injured ligament, or that of an intact ACL or PCL, could serve as a surrogate metric, enabling robust inferences about another grievously injured ligament’s diameter. By extrapolating these diameters to cross-sectional areas, clinicians can judiciously select grafts for reconstructive surgeries, ensuring superior restoration of knee functionality and bolstering patient outcomes.

In this investigation, we chiefly probed the interrelations between the diameters of typical ACLs and PCLs in sagittal MRI evaluations of standard knee joints. Traditional anatomical studies on three-dimensional cadavers, however, pose an inherent constraint to our project: the reliance on two-dimensional measurements. Given the inherently irregular morphology of each segment of the ACL and PCL [22–25], pinpointing the optimal point for diameter measurement within this plane proves challenging. Contemporary research brims with studies delving into the interplay between the cross-sectional areas of ACL, PCL, and grafts within the knee joint [26–28]. Yet, our approach – deriving diameters of the ACL and PCL from a two-dimensional plane – POSES hurdles in drawing correlations with the corresponding cross-sectional areas. This underscores the imperative for expanded studies, elucidating the ties between the diameters of ACLs and PCLs in standard human knee sagittal MRIs and their cross-sectional areas in conventional anatomical studies. Should a robust association between the diameter of each ligament’s point and its corresponding cross-sectional area be ascertained, one could deduce the latter simply by tracking diameter shifts in sagittal MRI examinations. Such a methodology would empower clinicians to swiftly and precisely pinpoint the requisite cross-sectional area, leveraging just the cruciate ligament diameter from a patient’s knee MRI. This study currently only includes gender as a variable, and in the future, further consideration will be given to incorporating factors such as age and weight.

## 5 Conclusion

In sagittal MRI evaluations of the right knee joint, the mean diameter at each location of the ACL surpassed that of its PCL counterpart. Male subjects consistently presented larger mean diameters than females across various anatomical landmarks, encompassing the PCL’s origin and midpoint as well as the ACL’s origin, midpoint, and insertion. Notably, the gender disparity in the PCL’s insertion point’s diameter lacked statistical significance in our analysis. Intriguingly, correlation analysis unveiled that only the PCL’s origin bore a moderate relationship with the ACL’s midpoint and insertion within standard knee joints in sagittal MRI of the right knee joint. Such insights could equip clinicians with a methodology to extrapolate and assess the diameter of a severely compromised cruciate ligament, leveraging data either from the remnant of an injured ligament or a relatively intact anterior cruciate ligament. This, in turn, could facilitate estimations of the cross-sectional area pertinent to each site, guiding the selection of optimal grafts for cruciate ligament reconstructive procedures.
